# Biogeography of Parasitic Nematode Communities in the Galápagos Giant Tortoise: Implications for Conservation Management

**DOI:** 10.1371/journal.pone.0135684

**Published:** 2015-09-02

**Authors:** Guillaume Fournié, Simon J. Goodman, Marilyn Cruz, Virna Cedeño, Alberto Vélez, Leandro Patiño, Caroline Millins, Lynda M. Gibbons, Mark T. Fox, Andrew A. Cunningham

**Affiliations:** 1 Galápagos Genetics Epidemiology and Pathology Laboratory, Galápagos National Park, Puerto Ayora, Santa Cruz, Galápagos Islands, Ecuador; 2 Ecole Nationale Vétérinaire d’Alfort, Maisons-Alfort, France; 3 Department of Production and Population Health, Royal Veterinary College, Hatfield, United Kingdom; 4 School of Biology, Faculty of Biological Sciences, University of Leeds, Leeds, United Kingdom; 5 Concepto Azul, Guayaquil, Ecuador; 6 Biotechnology Program, University of Guayaquil, Guayaquil, Ecuador; 7 Institute of Zoology, Zoological Society of London, London, United Kingdom; 8 Department of Pathology and Infectious Disease, Royal Veterinary College, London, United Kingdom; University of Pretoria, SOUTH AFRICA

## Abstract

The Galápagos giant tortoise is an icon of the unique, endemic biodiversity of Galápagos, but little is known of its parasitic fauna. We assessed the diversity of parasitic nematode communities and their spatial distributions within four wild tortoise populations comprising three species across three Galápagos islands, and consider their implication for Galápagos tortoise conservation programmes. Coprological examinations revealed nematode eggs to be common, with more than 80% of tortoises infected within each wild population. Faecal samples from tortoises within captive breeding centres on Santa Cruz, Isabela and San Cristobal islands also were examined. Five different nematode egg types were identified: oxyuroid, ascarid, trichurid and two types of strongyle. Sequencing of the 18S small-subunit ribosomal RNA gene from adult nematodes passed with faeces identified novel sequences indicative of rhabditid and ascaridid species. In the wild, the composition of nematode communities varied according to tortoise species, which co-varied with island, but nematode diversity and abundance were reduced or altered in captive-reared animals. Evolutionary and ecological factors are likely responsible for the variation in nematode distributions in the wild. This possible species/island-parasite co-evolution has not been considered previously for Galápagos tortoises. We recommend that conservation efforts, such as the current Galápagos tortoise captive breeding/rearing and release programme, be managed with respect to parasite biogeography and host-parasite co-evolutionary processes in addition to the biogeography of the host.

## Introduction

The Galápagos giant tortoise (*Chelonoidis nigra* species complex [1, 2]) is listed in CITES Appendix I and as vulnerable by the World Conservation Union (http://www.iucnredlist.org/details/9011/0) due to small population sizes, arising from a history of over exploitation for food, habitat loss and impacts from invasive species. Originally, Galápagos giant tortoise species were distributed across the archipelago [3, 4], all derived from the same South-American mainland lineage [5]. Today, and following the recent death of Lonesome George, the last known specimen of *C*. *abingdonii*, seven of ten recognised species [2] remain, with an estimated total population of between 6,000 and 20,000 individuals [6, 7] compared to around 250,000 prior to human arrival. Tortoises are present on six Galápagos islands: five islands (Santa Cruz, Santiago, Pinzón, San Cristobal and Espanola) each harbour one single-island endemic species, while one island (Isabela) harbours two single-island endemic species [2]. Although an icon of the unique Galápagos biodiversity, the Galápagos giant tortoise has been poorly studied and little is known of its parasitic fauna.

Parasitism is recognized as a fundamental factor driving the dynamics of wild animal populations. Through their impact on host fecundity and survival, parasites may regulate the size of their host populations and cause cyclic fluctuations [8, 9]. They constitute strong selective pressures for host genetic diversity and, therefore, influence the structure and the diversity of ecological communities and ecosystems [10]. Although parasites are important drivers of biodiversity, they can also present serious challenges for wildlife conservation, particularly when acting in conjunction with anthropogenic influences [11–13]. Assessing the composition and epidemiology of parasite communities of wild animal populations is, therefore, essential for understanding their conservation management.

There have been few studies on the gastro-intestinal nematodes of wild Galápagos giant tortoises [14–16]. Although parasitic nematodes have been described from wild Galápagos giant tortoises before [17], and have been implicated as a contributory cause of *C*. *porteri* mortality in two disease outbreaks on Santa Cruz island [18, 19], there are no baseline data against which to determine the impact of these parasites on tortoise populations. Given the diversification of the Galápagos tortoise lineage [20] across the archipelago, the composition of nematode communities might differ between various tortoise populations. This has not been considered to date, including by captive breeding and reintroduction programmes, even though this is likely to be important for the maintenance of genetic diversity and host adaptations. The introduction of novel nematode species into a tortoise population through, for example, conservation management interventions might disturb the composition of existing nematode communities [21].

Here, we report the diversity and variation in composition of parasitic nematode communities between and within Galápagos giant tortoise species. Our findings should inform decisions for the conservation of this overlooked component of Galápagos biodiversity and for the conservation management of the tortoise hosts.

## Materials and Methods

### Ethics Statement

This study was conducted with ethical approval from the Zoological Society of London’s Ethics Committee; project ref. WLE/0341. Additional permits were obtained from the Galápagos National Park.

### Sampled populations

**Fig 1:**
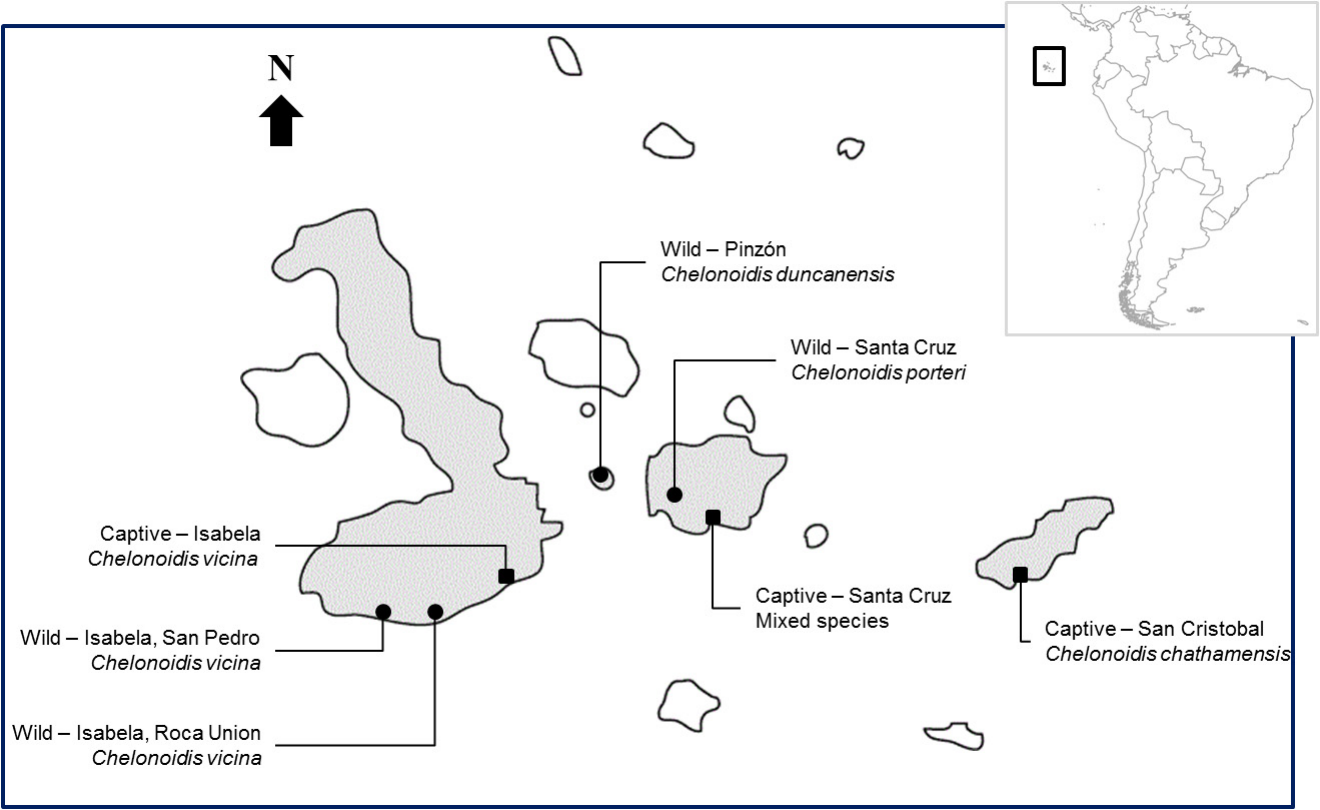
Map showing the locations where faecal samples were collected on the Galápagos Islands. Squares depict captive populations, circles depict wild populations. Inset—map of South America highlighting (in square) the location of the Galápagos Islands relative to the mainland.

We identified the presence of parasitic nematodes via microscopical examination of samples of freshly voided faeces. Faecal samples were collected between November 2005 and May 2006 from four wild tortoise populations (host species follows [2]): south-west Santa Cruz island (*C*. *porteri*), central Pinzón island (*C*. *duncanensis*) and Roca Union and San Pedro on Isabela island (*C*. *vicina)* (Fig 1). The sampling was opportunistic: the cloaca of each tortoise found was digitally stimulated, which often resulted in the voiding of fresh faeces. When this was successful, the age (adult, juvenile) and sex (male, female) of the tortoise was noted for each sample. Fresh (moist) faeces found on the ground were also collected.

In addition, adult tortoises were sampled in each of three captive breeding centres: on Santa Cruz (SXBC) (*C*. *abingdoni*, *C*. *hoodensis*, and undetermined species), Isabela (IBC) (*C*. *vicina*) and San Cristobal (SCBC) (*C*. *chathamensis*) islands. Also, four species (*C*. *porteri*, *C*. *duncanensis*, *C*. *hoodensis*, *C*. *darwini*) of juvenile tortoise destined for release into the wild were sampled from SXBC and one species (*C*. *vicina*) of juvenile tortoise was sampled from IBC. In the captive centres, adult tortoises were sampled via cloacal stimulation whilst faeces were collected from the ground of the enclosure housing the juvenile animals.

For both wild and captive tortoises, all faecal samples were stored at 4°C until they were examined up to a week post-collection.

### Faecal Egg Counts

A modified McMaster method (adapted from that described by Ministry of Agriculture, Fisheries and Food [22]) was used to assess faecal egg density. Briefly, 4.5g of material was taken from the core of each faecal sample, suspended in 25 mL of saturated sodium chloride (NaCl) solution and poured through a 1mm sieve to remove any large debris. Subsequently, 15.5 mL of saturated NaCl solution was added and the mixture was agitated to obtain a homogeneous suspension. A 0.3 mL aliquot of the filtrate was removed using a Pasteur pipette. It was placed immediately into two chambers of a McMaster slide, with each of them representing 0.15 mL. Hence, if two chambers are counted, the count represents the amount of eggs in 0.3 mL suspension. After 2 min, any eggs present in each chamber were counted under a light microscope at 100× magnification. The number of eggs per gram of faeces (epg) was obtained by multiplying the combined number of eggs counted in both chambers by 10. Duplicate egg counts were conducted on 250 faecal samples in order to test the repeatability of the method.

The examinations of the tortoise faecal samples were conducted in the field on the Galápagos archipelago without access to a graticule, therefore quantitative data on egg size could not be obtained. The relative sizes of the eggs, however, were noted. A representative sample of egg morphotypes was photographed with a scale bar on return to the laboratory.

### Statistical Analysis

The degree of repeatability of the egg counting method was tested by calculating the Pearson’s correlation coefficient [23] for the results obtained from duplicate faecal egg counts from 250 samples.

We calculated the proportion of parasitized tortoises, plus associated confidence interval by the exact Sterne method [24]), the mean abundance (arithmetic mean number of eggs per tortoise, plus associated confidence interval calculated by bootstrap [25]), and two parameters of aggregation, the variance to mean ratio and the *k* parameter of the negative binomial distribution [26], for each nematode egg type from each of the tortoise populations examined. The *k* parameter was estimated by maximizing the log-likelihood function derived from the probability mass function of the negative binomial distribution, and the confidence interval was estimated as described in [27]. The goodness of fit to a negative binomial distribution was assessed by computing the likelihood ratio between saturated and negative binomial models (sample size > 50), or by using a q-q plot (for lower sample sizes).

The proportions of parasitized tortoises were compared between populations using Fisher’s exact test. When comparing mean egg abundances between several groups (i.e. more than 2 groups), negative binomial generalized linear models (GLM) were used [28]. When comparing mean egg abundances between two groups, a Bootstrap *t* test where the null distribution of the *t* statistic was determined by bootstrapping was used due to the over-dispersion of the data [29]. For each GLM, the likelihood ratio (LR) between the specified and the null model, and its associated p-value were reported. A population was chosen as reference (or contrast) and differences in egg abundance between this reference population and others were assessed using the Wald test. Z-scores and p-values were reported. Multiple tests were performed using Bootstrap *t* tests [29]. A Bonferroni correction was applied. The estimation of the k parameter and the fitting of the GLMs were conducted using the number of eggs counted in both McMaster chambers (i.e. number of eggs per 0.1 gram of faeces). Within a population, the proportions of tortoises parasitized by two different egg types were compared using an exact binomial test, also known as an exact McNemar test. In practice, the number (S) of tortoise parasitized by type *i* but not *j* was compared to a binomial distribution with parameters the number of tortoises parasitized by only one (*i* or *j*) of the two types (T), and a probability of success of 0.5. As all possible combinations of egg types were compared, a Bonferroni correction was also applied. Number of successes and trials (S/T) and associated p-values are reported.

Within a population, we compared proportions and means, between sex or age sub-groups, by using Fisher’s exact test and a bootstrap *t* test, respectively. Sex and age categories were not included as covariates of GLMs because this information was only available for a small number of tortoises. There is some evidence that the number of parasites infecting a host can be positively correlated with its body size in several host-parasite systems [30]. To assess if there was a body size bias, we calculated the Spearman’s correlation coefficient with the associated p-value, assuming a positive correlation between the number of nematode eggs per gram of faeces and the number of nematodes parasitizing each tortoise. The body size of a tortoise was here defined as the carapace length.

All analyses were conducted using the R software package Version 2.15.2 (www.r-project.org).

### Species assignment of expelled nematodes

Tortoises were observed to occasionally expel nematodes in their faeces. When present, these were identified using morphological characters and genetic analysis. Morphological identification was conducted using light microscopical examination of temporary slides of the nematodes, which were made using lactoglycerol as the mounting and clearing agent. For genetic analysis, DNA was extracted from adult nematodes using a standard NaOH protocol [31]. A 500bp section of the 18S ribosomal RNA gene, commonly used in phylogenetic assessment of nematodes [32], was amplified using universal 18S primers 18SF (5' CGC GAA TRG CTC ATT ACA ACA 3') and SSU_R_09 (5' AGC TGG AAT TAC CGC GGC TG 3') (positions 111–123 and 565–584 respectively with reference to the *Caenorhabditis elegans* 18S gene) ([33]; www.nematodes.org/barcoding/sourhope/nemoprimers.html). PCR was carried out in a total volume of 50 μL with 10x PCR reaction buffer (Invitrogen), 0.25 mM each dNTP, 5.0 mM MgCl_2_, 2 pmol/μL each primer, 5 u/μL Taq polymerase (Invitrogen), and approximately 50 ng of DNA template. The PCR temperature cycle consisted of: 94°C for 5 minutes; 35 cycles of 94°C for 30s, 52°C for 45s and 72°C for 45 seconds; with a final extension cycle of 72°C for 7 minutes. PCR products were sent for sequencing with the commercial service provider Macrogen (South Korea). Bioedit [34] was used to process the raw sequence data, and Clustal W, implemented in Bioedit, was used to generate an alignment of the Galápagos nematode sequences with representative 18S sequences from a range of nematode species from the known nematode families, imported from Genbank. The Galápagos sequences were assessed by BLAST comparison [35] against the Genbank sequence database. Phylogenetic analysis and drawing of the resulting phylogenies was carried out using MEGA v4.1 [36]. We used the Kimura 2-parameter genetic distance and Neighbour Joining to generate the tree. Bootstrapping with 1000 replicates was used to assess support for the topology of the tree (S1 Fig).

## Results

Three-hundred-and-sixty-two tortoise faecal samples were collected and examined for parasite eggs: 207 from wild animals and 155 from captive animals. The number of samples collected from each tortoise population examined, with an indication of the size of each of these populations, is presented in Tables 1 and 2. In the following, nematode communities refer to the taxonomic diversity of nematode eggs within each tortoise population, described at the superfamily level.

**Table 1:** Wild Galápagos giant tortoise (*Chelonoidis* spp.) faecal samples collected and the results of parasitological examinations.

Island	Santa Cruz	Isabela		Pinzón
Tortoise species	*C*. *porteri*	*C*. *vicina*		*C*. *duncanensis*
Site	South-West	Roca Union	San Pedro	Centre
Population size	~ 3000	~ 100	~ 400	~ 500
Sample size	126	25	22	34
Adults^a^	55	13	8	30
Females^a^	16	11	0	20
Males^a^	39	2	8	10
Juveniles^a^	5	10	11	4
Nematode eggs				
Overall–Prop (%) (CI 95%)	81.7 (73.9–88.1)	92 (74–99)	81.8 (59.7–94.8)	88.2 (72.5–96.7)
Trichurid–Prop	0.8 (0–4.3)	20 (6.8–40.7)	4.5 (0.1–22.8)	-
Mean epg (CI 95%)	<1	4 (1–8)	<1	-
*S* ^*2*^ */m*	20	21	10	-
*k* (CI 95%)	-	-	-	-
Ascarid–Prop	8.7 (4.4–15.1)	12 (2.5–31.2)	9.1 (1.1–29.2)	32.4 (17.4–50.5)
Mean epg	1 (1–2)	2 (0–5)	1 (0–2)	6 (3–10)
*s* ^*2*^ */m*	16	21	10	22
*k*	-	-	-	0.5 (0.2-∞)
Oxyuroid–Prop	11.1 (6.2–17.9)	8 (1–26)	4.5 (0.1–22.8)	-
Mean epg	2 (1–3)	2 (0–4)	1 (0–4)	-
*s* ^*2*^ */m*	25	19	30	-
*k*	-	-	-	-
Small-strongyle–Prop	-	88 (68.8–97.5)	81.8 (59.7–94.8)	-
Mean epg	-	404 (153–768)	54 (33–77)	-
*s* ^*2*^ */m*	-	1761	54	-
*k*	-	0.4 (0.3–0.8)	0.9 (0.5–3.7)	-
Large-strongyle–Prop	80.2 (72.1–86.7)	12 (2.5–31.2)	9.1 (1.1–29.2)	88.2 (72.5–96.7)
Mean epg	78 (56–104)	4 (0–12)	1 (0–2)	77 (52–105)
*s* ^*2*^ */m*	255	61	10	85
*k*	0.5 (0.4–0.7)	-	-	1 (0.6–2.2)
Undefined–Prop	5.6 (2.3–11.1)	8 (1–26)	-	-
Mean epg	1 (0–1)	1 (0–3)	-	-
*s* ^*2*^ */m*	14	16	-	-
*k*	-	-	-	-

a. Age and sex were not available for all samples. Prop: proportion of parasitized tortoises. epg: number of nematode eggs counted per gram of faeces (CI 95%): 95% confidence interval for the proportion, the mean and the *k* parameter. *k*: parameter of overdispersion of negative binomial distribution. *s* ^*2*^ */m*: variance to mean ratio.

**Table 2:** Captive Galápagos giant tortoise (*Chelonoidis* spp.) faecal samples collected and the results of parasitological examinations.

Island	Santa Cruz		Isabela		San Cristobal
Tortoise species	Mixed^a^		*C*. *vicina*		*C*. *chathamensis*
Site	Adult enclosures	Juvenile enclosures	Adult enclosures	Juvenile enclosures	Adult enclosures
Population size	75	673	*Unknown*	*unknown*	37
Sample size	65	32	21	10	27
Nematode eggs					
Overall–Prop (%) (CI 95%)	58.5 (45.6–70.6)	25 (11.5–43.4)	9.5 (1.2–30.4)	10 (0.3–44.5)	-
Trichurid–Prop	-	-	-	-	-
Mean epg (CI 95%)	-	-	-	-	-
*s* ^*2*^ */m*	-	-	-	-	-
*k* (CI 95%)	-	-	-	-	-
Ascarid–Prop	6.2 (1.7–15)	-	4.8 (0.1–23.8)	-	-
Mean epg	1 (0–1)	-	<1	-	-
*s* ^*2*^ */m*	10	-	10	-	-
*k*	-	-	-	-	-
Oxyuroid–Prop	1.5 (0–8.3)	-	-	-	-
Mean epg	<1 (0–1)	-	-	-	-
*s* ^*2*^ */m*	30	-	-	-	-
*k*	-	-	-	-	-
Small-strongyle–Prop	-	-	9.5 (1.2–30.4)	-	-
Mean epg	-	-	13 (0–37)	-	-
*s* ^*2*^ */m*	-	-	198	-	-
*k*	-	-	-	-	-
Large-strongyle–Prop	58.5 (45.6–70.6)	25 (11.5–43.4)	9.5 (1.2–30.4)	10 (0.3–44.5)	-
Mean epg	34 (23–46)	5 (1–11)	10 (0–30)	2 (0–6)	-
*s* ^*2*^ */m*	69	42	190	20	-
*k*	0.4 (0.3–0.8)	-	-	-	-
Undefined–Prop	-	-	-	-	-
Mean epg	-	-	-	-	-
*s* ^*2*^ */m*	-	-	-	-	-
*k*	-	-	-	-	-

a. In the Santa Cruz breeding centre, sampled adult tortoises comprised 1 *C*. *abingdoni* (from Pinta island), 20 *C*. *hoodensis* (from Espanola island) and 44 of undetermined species; sampled juveniles belonged to the species *C*. *porteri*, *C*. *duncanensis*, *C*. *hoodensis* and *C*. *darwini*. epg: number of nematode eggs counted per gram of faeces (CI 95%): 95% confidence interval for the porportion, the mean and the *k* parameter. *k*: parameter of overdispersion of negative binomial distribution. *s* ^*2*^ */m*: variance to mean ratio.

### Diversity of nematode egg types

**Fig 2:**
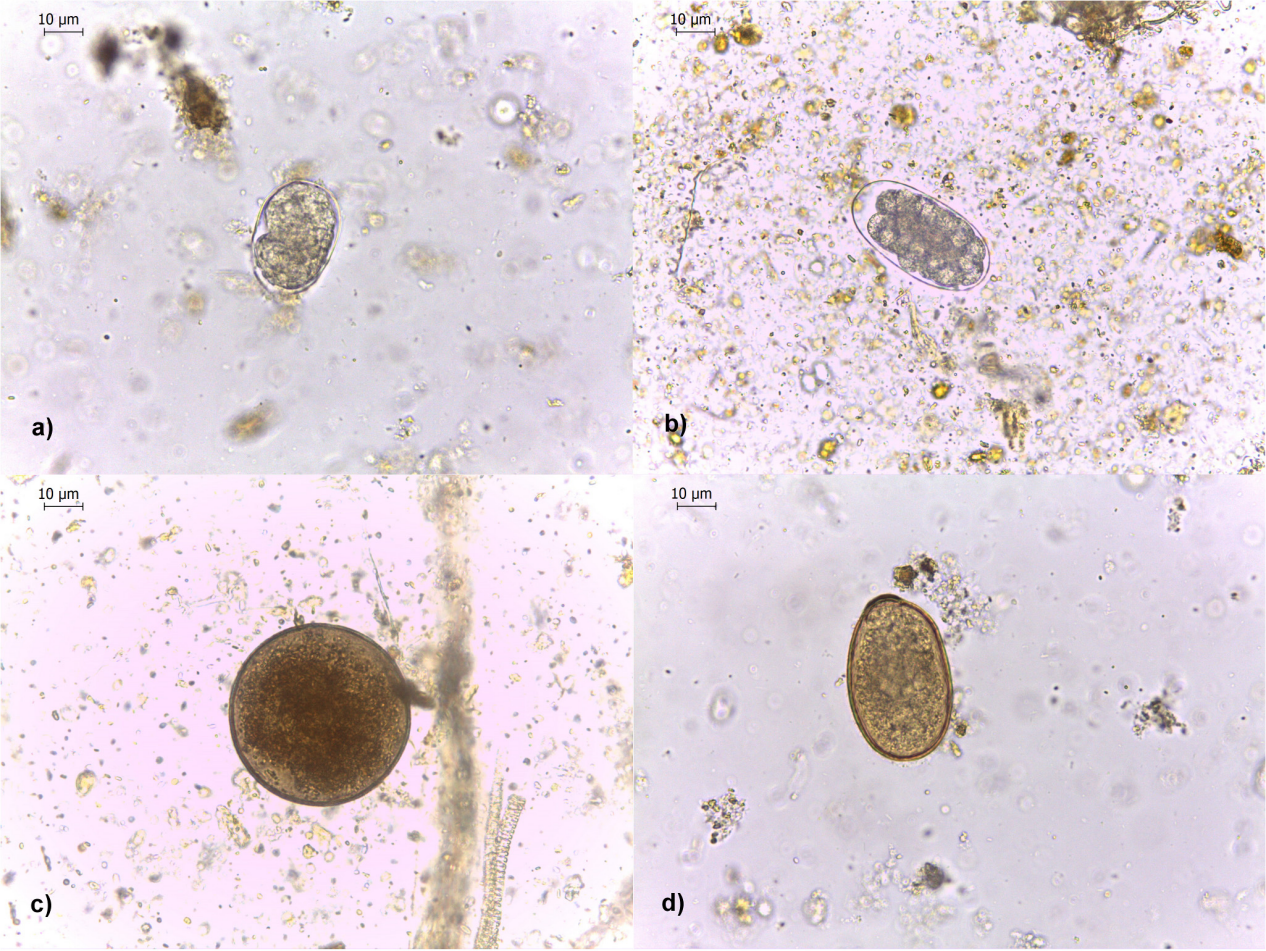
Photomicrographs of nematode eggs found in Galápagos giant tortoise faecal samples. (a) Small strongyle egg. (b) Large strongyle egg. (c) Ascarid egg. (d) Oxyurid egg. N.B. No trichurid eggs were found in samples returned to the laboratory for photography.

**Fig 3:**
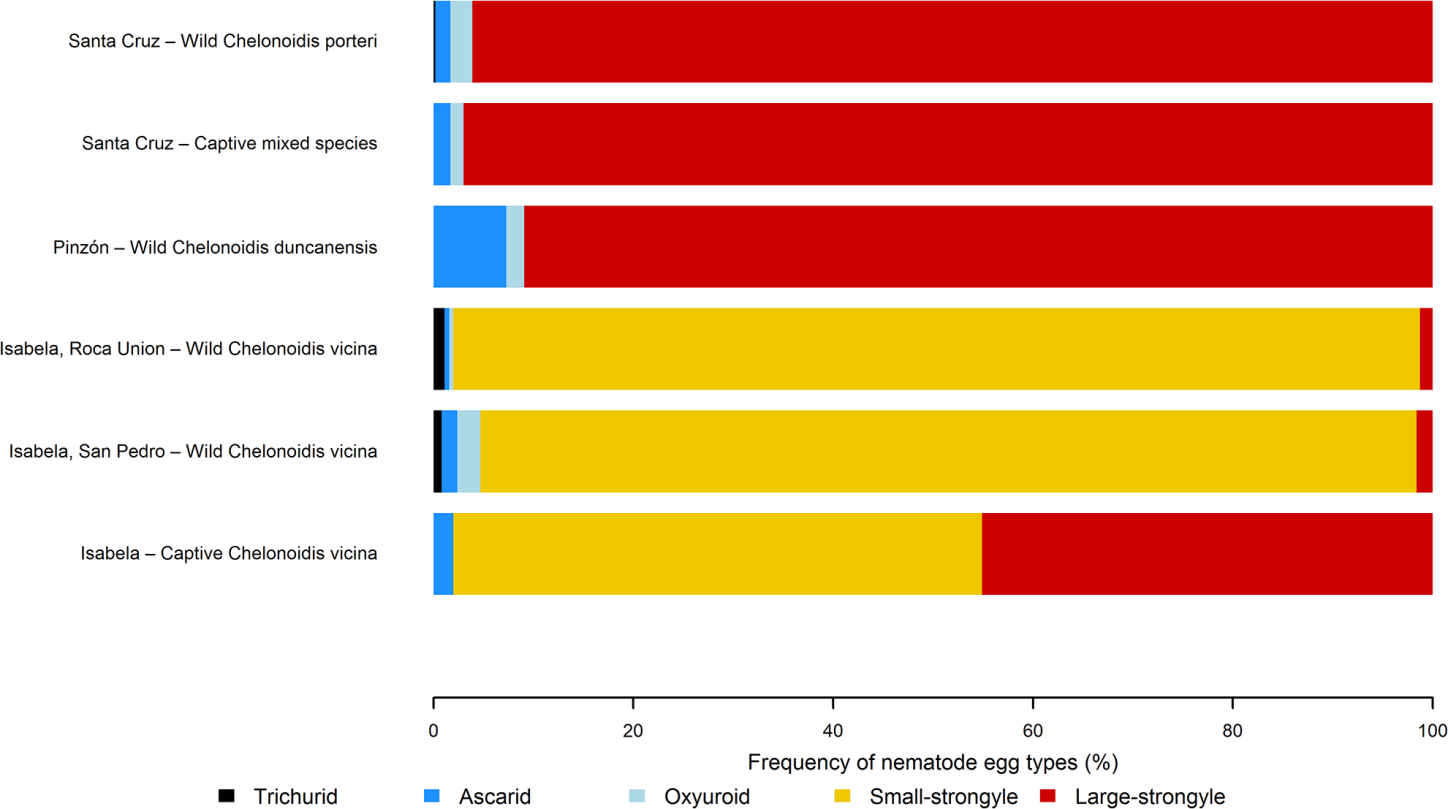
Relative frequency of nematode egg types according to location. Relative frequencies are expressed as a percentage.

Nematode parasite eggs were found in faeces from every tortoise population examined except for captive tortoises from SCBC, but the composition of nematode communities differed with tortoise species, which co-varied with island. We identified five distinct types of nematode egg representing four different nematode superfamilies (Strongyloidea, Ascaridoidea, Trichinelloidea and Oxyuroidea) (Fig 2; S1 Table). Tortoises from all of the wild populations examined had strongyle, oxyuroid and ascarid eggs in their faeces, whilst trichurid eggs were present in all wild populations of tortoise except for the one on Pinzón island (*C*. *ducanensis*). Two types of strongyle egg were identified, based on size: a “large” type (Fig 2) which was approximately twice the size of a “small” type (Fig 2). The distributions of the two types of strongyle were clearly heterogeneous: large-strongyle eggs were found in all wild tortoise populations examined, whereas small-strongyle eggs were found only in the wild populations of San Pedro and Roca Union on Isabela Island, where they were predominant (Fig 3).

In the captive populations examined, large-strongyle, oxyuroid and ascarid eggs were detected from tortoises held at the SXBC; ascarid, small-strongyle and large-strongyle eggs were detected from tortoises at the IBC. In the SXBC, faecal samples from captive-bred juveniles, which are housed separately from the adults, contained only strongyle eggs.

Within each tortoise population, the relative frequencies of nematode types did not differ with either the age or the sex of the host.

### Faecal egg counts

The egg counting method employed gave a very high degree of repeatability, with a Pearson’s correlation coefficient of 0.93. Generally, egg counts were low for each tortoise population and for each nematode type when compared to those reported from tortoises elsewhere [37], with mean counts ranging from 1 to 404 epg, when present (Tables 1 and 2). In all tortoise populations examined, strongyle eggs accounted for more than 90% of counted eggs (Fig 3).

### Species assignment of expelled nematodes

**Fig 4:**
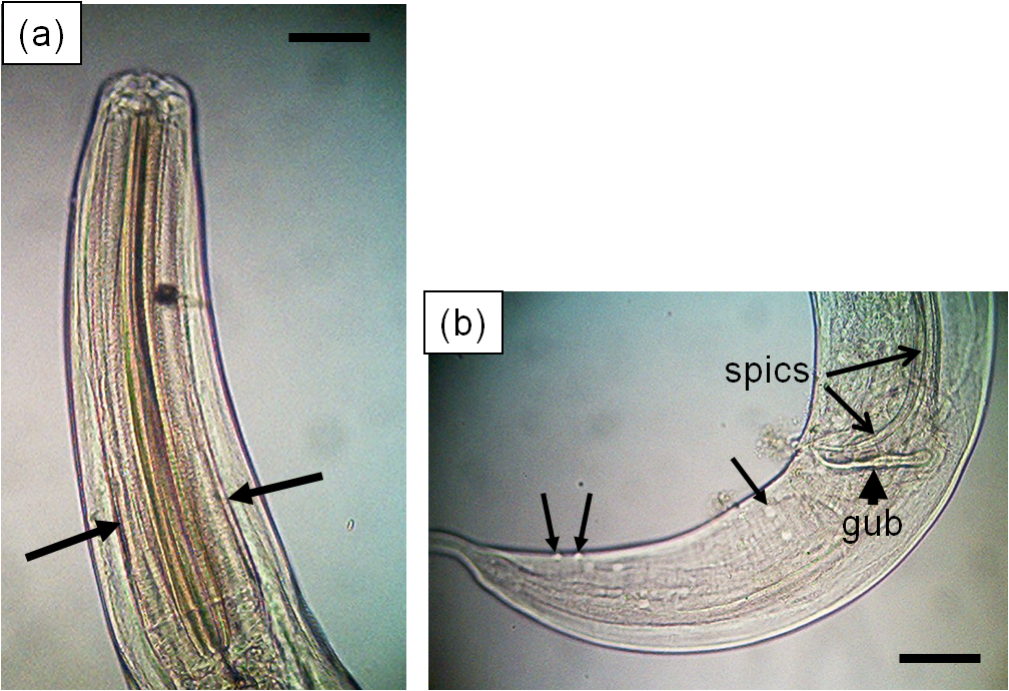
Photomicrographs of a male *Atractis* sp. nematode detected in a Galápagos giant tortoise faecal sample. Scale bars = 50 μm. (a) Anterior end of body showing wider sclerotised anterior region of the oesophagus [or pharynx] (arrows). (b) Lateral view of posterior end of male showing caudal papillae (closed arrows), gubernaculum (arrowhead) and distal ends of spicules (open arrows).

**Fig 5:**
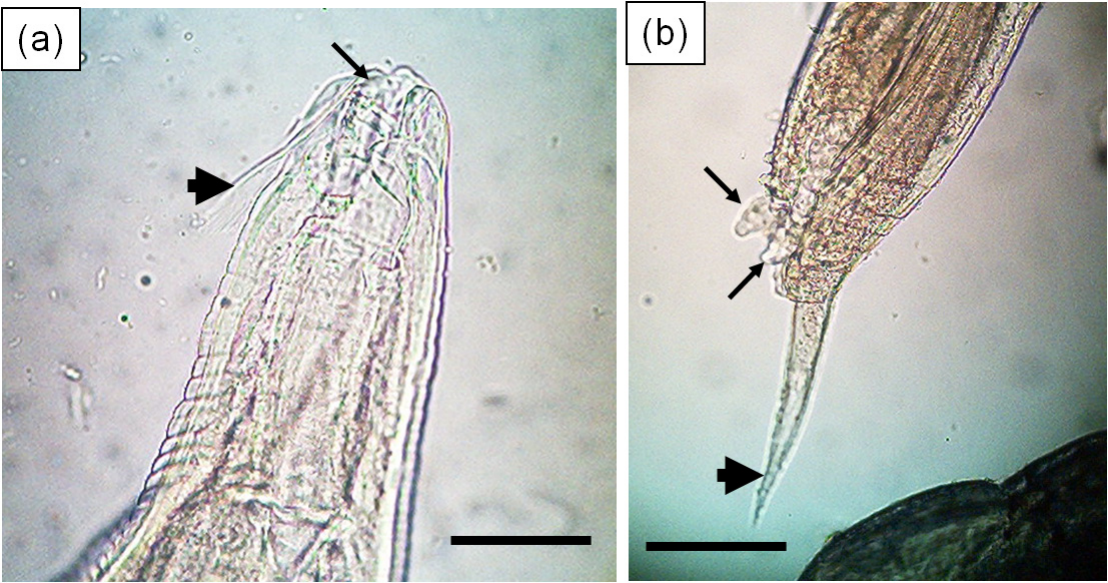
Photomicrographs of a male *Labiduris* sp. nematode detected in a Galápagos giant tortoise faecal sample. Scale bars = 50 μm. (a) Anterior end showing the subventral lips (arrow) with a posteriorly-directed fringe along the median edge (arrowhead). (b) Posterior end showing terminal appendix (arrowhead) and ventro-lateral papillae (arrows).

Thirty-two adult nematodes were obtained from tortoise faecal samples collected from *C*. *porteri* in Santa Cruz. When observed using a light microscope, 30 nematodes were found to belong to the genus *Atractis* (Fig 4) and the remaining two, to the genus *Labiduris*, (Fig 5) based on a suite of standard morphological characters [38, 39]. Following the classification of Chabaud [40], both genera are placed in the family Atractidae (Ascaridida, Cosmocercoidea). 18S small subunit sequences were obtained from 11 specimens of *Atractis* sp. and from both of the *Labiduris* sp (Royal Veterinary College collection, accession number: 8064). The two morphological types yielded two distinct sequences with approximately 94.4% sequence identity. These are deposited in Genbank with accession numbers KT364749 and KT364750 for *Atractis* and *Labiduris*, respectively. In each case there was no direct match with BLAST against the Genbank database suggesting that these sequences come from previously-unsequenced nematode species. With BLAST and the phylogenetic analysis, the sequences from *Atractis* sp. clustered most closely with *Rondonia rondoni (Ascaridida*, *Atractidae)*, a nematode found in South American tropical freshwater fish (Genbank accession: DQ442679; 96% sequence identity, 97% bootstrap support (S1 Fig)). The next closest match, at 93% sequence identity, was with *Thelastoma gueyei* (Oxyurida, Thelastomatidae) (Genbank accession: AM260939). The sequence from *Labiduris* sp. exhibited ~96% sequence identity and 66% bootstrap support, with *Cucullanus* (*Truttaedacnitis*) *truttae* (Ascaridida, Cucullanidae) (Genbank accession: EF180063), and 94% sequence identity with *Teratocephalus lirellus* (Teratocephalidae) (following the classification of De Ley & Blaxter [41] this genus is placed in the Rhabditida, but the position of the family Teratocephalidae is considered *incertae sedis*) (Genbank accession: AF036607).

### Nematode egg distributions and abundances in wild Galápagos tortoises

All wild populations of tortoises examined had nematode eggs present, with the proportion of tortoises parasitized with strongyles being higher than 80% in all examined wild populations (Table 1). Within each population, the proportion of tortoises parasitized by strongyle eggs–large type in Santa Cruz and Pinzon, small type in Roca Union and San Pedro–was higher than for any other nematode superfamily (Exact binomial test, all p-values<0.001; Santa Cruz: Trich (S = 100/T = 100), Asc (90/90), Oxyur (88/89); Pinzon: Asc (19/19); Roca Union: Trich (18/19), Asc (19/19), Oxyur (20/20), Large St (19/19); San Pedro: Trich (17/17), Asc (16/16), Oxyur (17/17), Large St (16/16)).

The mean abundances of large-strongyle infection were location-dependent (GLM: LR, χ^2^_3_ = 88, p-value<0.001), with the mean abundance of large-strongyle eggs in Roca Union and San Pedro populations being significantly lower than that in the Santa Cruz (z = -6.7 and -5.7, p-value<0.001, respectively) and Pinzón populations (bootstrap *t* test: p-value<0.001). While small-strongyle eggs were only observed in Isabela, their mean abundance was higher in Roca Union than in San Pedro (bootstrap *t* test: p-value<0.01). The abundance of ascarid eggs was higher in Pinzon, than in Santa Cruz and San Pedro (GLM: LR, χ^2^_2_ = 15, p-value<0.001; z = -3.5 and -2.2, p-value<0.001 and 0.03, respectively), and the abundance of trichurid eggs was higher in Roca Union than in Santa Cruz (Generalized linear model: LR, χ^2^_2_ = 17, p-value<0.001; z = -3.4, p-value<0.001).

### Nematode egg distributions according to sex and age in wild Galápagos tortoises

Due to sample size limitations, the effects of age and sex were only explored in the San Pedro and Roca Union populations, and in the Santa Cruz and Pinzón populations, respectively. Neither proportion of parasitized tortoises nor mean abundance varied with age category (Fisher’s exact test p-value = 0.1, 0.56; Bootstrap *t* test p-value = 0.45, 0.07 for small-strongyle eggs in San Pedro and Roca Union, respectively) or with sex (Fisher’s exact test p-value = 0.66, 0.53; Bootstrap *t* test p-value = 0.06, 0.69 for large-strongyle eggs in Santa Cruz and Pinzón, respectively) (Table 3).

**Table 3:** Description of nematode egg distributions according to sex or age class within four wild populations of Galápagos giant tortoise.

Island	Santa Cruz		Isabela				Pinzón	
Tortoise species	*C*. *porteri*		*C*. *vicina*				*C*. *duncanensis*	
Site	South-West		Roca Union		San Pedro		Centre	
Sex/Age class	Males	Females	Adults	Juveniles	Adults	Juveniles	Males	Females
Sample size	39	16	13	10	8	11	10	20
Counted eggs								
Prop (%)	84.6	93.8	92.3	80	100	63.6	100	85
(CI 95%)	(69.5–94.1)	(69.8–99.8)	(64–99.8)	(44.4–97.5)	(63.1–100)	(30.8–89.1)	(69.2–100)	(62.1–96.8)
Mean epg	63	158	186	748	64	45	91	78
(CI 95%)	(39–91)	(63–290)	(85–300)	(166–1590)	(29–102)	(15–78)	(32–172)	(52–106)
*S* ^*2*^ */m*	113	377	225	2163	52	69	149	52

*s* ^*2*^ */m* = variance to mean ratio.

There was no significant correlation between host body size and faecal egg count, discounting the possibility of a body size effect on the nematode egg output (for Santa Cruz, Pinzón, Roca Union, and San Pedro, n = 27, 34, 23, 19; Spearman Coefficient ρ = -0.19, -0.01, -0.09, 0.24; p-value = 0.33, 0.95, 0.69, 0.33; respectively).

### Nematode egg distributions and abundances in captive Galápagos tortoises

The proportion of parasitized tortoises and parasite egg abundances were lower among captive than wild populations, in particular among juveniles (Table 2). Excluding adults in SXBC, where over half the population was positive for nematode eggs, the proportion of captive tortoises found to be infected was always less than 25%. No parasite eggs were found in faecal samples from SCBC, and only two of 21 adults in IBC had nematode eggs.

## Discussion

The examination of nematode eggs in tortoise faeces revealed the presence of five types of nematode to be infecting tortoises on the Galápagos archipelago, comprising four superfamilies (Strongyloidea, Ascaridoidea, Trichinelloidea and Oxyuroidea). Two types of strongyle were identified. In addition we identified at least two species of ascarid nematodes (*Atractis* sp. and *Labiduris* sp.) based on morphological characteristics and 18S small subunit ribosomal RNA sequences. Prior to this study, the only nematode described from tortoises in Galápagos was the ascarid *Atractis marquezi* [39]. It is likely that the *Atractis* sp. found in the current study is *A*. *marquezi* as this nematode was found in the same Santa Cruz *C*. *porteri* population examined by Bursey & Flanagan [39]. *Atractis* sp. and *Labiduris* sp. were not represented in the egg counts, as Atractidae eggs hatch and larvae develop in utero [42]. To the authors’ knowledge, this is the first DNA sequence data for these two genera.

In all of the wild tortoise populations examined, nematode infections were common, with more than 80% of tortoises being infected, and strongyle eggs were the most abundant in these populations, accounting for > 90% of eggs counted. The high abundance of strongyle eggs might suggest that strongyle nematodes are the most abundant nematode in Galápagos tortoises, however, faecal egg counts might not give a true representation of parasite burden: it was not possible to assess the relation between egg counts and worm burdens. Generally, egg counts were low across all populations and for all nematode types. This might be due to low worm burdens or to low fecundity of parasites [37].

Nematode communities in other tortoise species are known to be highly diverse [43]. The term “nematode communities” as used here refers to the taxonomic diversity of nematode eggs at the superfamily level. As an egg type may correspond to several nematode species, the patterns that we presented may change once the composition of these communities are described at the species level. Quantification of egg sizes may have helped to further differentiate egg types and identify variations in nematode community compositions. Moreover, we did not definitively assess the diversity of Galápagos tortoise gastro-intestinal nematode communities: low sample sizes and the low sensitivity of the McMaster egg counting method [44] could cause an underestimation of diversity. For instance, the sample size in Pinzon (n = 34) meant that we could detect with a probability of 95% the presence of a given nematode type in this tortoise population only if its true prevalence was higher than 8.5%. In addition, collecting only one faecal sample per tortoise and the low sample size associated with high parasite aggregation patterns could lead to an underestimation of real infection levels [45, 46]. These potential limitations should be taken into account when interpreting our results.

### Nematode diversity in Galápagos tortoise populations

The composition of nematode communities varied according to tortoise species, which co-varied with island. While sampling effects in the study may have influenced the observed composition of the investigated nematode communities, and especially the presence or absence of rare egg types, evolutionary and ecological factors may also be responsible for some of this variation. The *Chelonoidis nigra* lineage is monophyletic [5], therefore, assuming that their nematodes are specific to tortoises, it seems likely that all of the nematode types we identified colonised Galápagos together with the founding host population. The different compositions of nematode communities on different islands might result from subsequent island colonization, as well as vicariance events [20]. As a null model we can consider that differences in community composition arose from a neutral process of drift and founder events during colonisation of the archipelago by tortoises, causing shifts in frequency or loss of nematode species in some species. Additionally, speciation of nematodes could occur in isolated tortoise populations (but it is unlikely that this would be detected from the egg morphology used in this study). The current nematode faunas on each island might be a subsample of a more-diverse ancestral community, with varying ecological conditions on each island, or competitive interactions between nematode species creating evolutionary pressures influencing community composition. The present nematode diversity in tortoise populations thus likely reflects the evolutionary history of these host-parasite interactions [47, 48]. Distinguishing the relative roles of these different processes in shaping the parasite community structure we see today requires further work, but Galápagos giant tortoises may provide an interesting system for studying the development of multi-parasite community structure.

The host spectrum of the nematodes identified in this study, however, is unknown, and could potentially include other species than *C*. *nigra*. The host specificity of nematodes parasitizing reptiles is variable: some nematode species can be specialized to a single host species, while others can parasitize several host species [49]. To the authors’ knowledge, while some nematode species are known to parasitize several host species within the Chelonii order [15, 43, 50], there is no evidence in the literature of tortoise nematodes parasitizing other reptile orders. However, since ascarid, oxyurid and strongyle nematodes are frequently found in a wide range of reptile species [51], the potential cross-species transmission of the nematodes we observed in Galápagos tortoises, with, for instance, sympatric lava lizards (*Tropidurus* spp.), while unlikely, cannot be excluded at this time.

### Patterns of nematode abundance in Galápagos tortoise populations

Although > 80% of tortoises were parasitized in each of the wild populations examined, nematode egg abundance and aggregation varied according to location. Nematode distributions are known to be highly influenced by ecological factors [47] which can modify parasite survival in the environment, and thus the level of exposure of hosts, and can influence host fitness and, hence, susceptibility to infection. The wild tortoises in Santa Cruz were sampled in the humid vegetation zone which comprised dense, shaded woodland with high rainfall. Here, the tortoise diet is diverse and includes grasses and fruit. In contrast, the wild tortoises sampled in Pinzón and in both locations in Isabela (Roca Union and San Pedro) were sampled in the dry vegetation zone where the environment is harsh and arid with sparse, primarily cactus, vegetation.

Large-strongyle eggs were found in all wild tortoise populations examined and they were always the most abundant nematode egg found except on Isabela, the only island where small-strongyle eggs were found and where these eggs predominated. This pattern of relative frequency might be explained by a competitive interaction and within-host density-dependent effects between the two types of strongyle nematode [52, 53]. Since eggs, and not actual parasites, were counted and as neither egg type could be identified to the species level, this hypothesis requires further investigation in order to be tested.

Although several studies have shown that sex and age may impact on the level of parasitism [45, 54, 55], no difference in egg abundance was observed according to age or sex in this study. The low sample sizes, however, do not allow us to definitively conclude about the presence or absence of such an impact. To do this, further studies measuring the abundance of nematodes, if possible at the species level, and for a larger fraction of these populations, are required.

### Implication for conservation programs

In this study, we were able to identify nematode eggs only to the superfamily level, therefore the apparent similarities in the composition of nematode communities between San Pedro and Roca Union tortoise populations, or between Pinzón and Santa Cruz populations should be taken cautiously. Indeed, describing nematodes at the species level may reveal variations in the composition of these communities that are not apparent at the current level of resolution. It is likely that nematode parasites have diversified genetically among isolated tortoise populations, leading to unique evolutionary and ecological parasite-host-location association. Although the specificity of this association remains uncertain, and the likelihood of allopatric evolutionary processes, and co-evolution of nematodes with their hosts, should be explored [56, 57], the precautionary principle dictates that it should be considered in conservation programs, and steps should be taken to avoid potential mixing of parasites between distinct tortoise populations and species.

In breeding centres, juvenile tortoises from several islands can be held together within the same enclosure and the population density is high. Additionally, no biosecurity measures are in place to keep juveniles free of indirect infection from adults and the risk of cross-infection from adults to juveniles is high. Juvenile tortoises are released to the islands from which their species originates, but no consideration is given to parasite biogeography and its maintenance. At the time of writing, there are no procedures in place to examine tortoises for parasite infection prior to release, or to act on the findings of these procedures if they are carried out. It is possible, therefore, that on release, juvenile tortoises co-introduce parasite species which have been acquired in captivity and which are not present in the wild tortoises at the site (or on the island) of release.

Galápagos giant tortoises have been subject to an intensive captive breeding and translocation program over the past three decades [58]. For example, 552 tortoises have been released on Pinzón island where the original surviving population was estimated at less than 100 individuals [6, 59]. All tortoises on Espanola have been bred in SXBC, or are offspring of tortoises bred in this centre [59]. In addition to potentially disrupting millennia of host-parasite co-evolution and parasite biogeography, the impact of the release program on tortoise population demography should be considered. Although nematodes have likely co-evolved with their hosts, the assumption that host-parasite co-evolution will tend towards avirulence is misleading [56]. Ecological changes and novel host-parasite or parasite-parasite interactions can lead to changes in host or parasite dynamics and could alter the impact of parasites on their hosts. Furthermore, small and inbred host populations with reduced genetic variability could have an increased susceptibility to new parasites [60].

Such issues as the release of immunologically naive animals into areas where parasites are endemic and the potential co-introduction of novel parasites with animals released for conservation reasons have been recognised previously [61, 62] and recommendations to minimise the impact of such conservation release programs on host-parasite interactions have been published (e.g. [61]). Management protocols should be implemented to ensure the separation of evolutionarily distinct host-parasite associations. This could include the separation of species within captive breeding centres with appropriate biosecurity and parasite monitoring protocols to minimise the risk of inter-species infections. Also, steps, such as anthelmintic treatment with follow-up faecal egg count monitoring, should be considered for tortoises prior to release. The actual designs of these management protocols and possible anthelmintic treatment procedures should be informed by further investigations of the diversity of nematode communities at a lower taxonomic level. While the current results suggest that the composition of nematode communities vary across tortoise populations, the practical conservation actions to be taken should be decided based on a higher-resolution understanding of the nematode communities. Indeed, the described nematode community patterns may differ at the species level.

### Future research directions

The results of this study need to be followed-up with further sampling to establish the species and island-specific nature of the tortoise nematode communities reported here. Also, research using more-detailed molecular analysis is required to investigate if nematodes of the same superfamily from different islands are evolutionary distinct, as we have hypothesised here. Extending the molecular analysis of Galápagos giant tortoise nematodes will allow exploration of the potential co-evolution between these parasites and their host(s). It may also give deeper insights into the biogeography and evolution of the tortoises, since the faster life-history of nematodes may record events not captured by the phylogenetic signals present in the genomes of the tortoises themselves. The life cycle of these parasites and their host spectrum should also be explored. The *post mortem* investigation of all tortoises that die in the captive breeding centres is also recommended as this would assess the presence of endemic or alien nematodes, along with their contribution, if any, to host mortality. Searching for other macroparasites, as well as microparasites, in dead and live tortoises would allow a broader assessment of the potential pathogens that could threaten wild tortoise populations.

The conservation of endangered species should not just be focused on the protection of specific species populations, but should also target the protection of their ecological communities and inter-specific interactions including co-evolved parasites. While this might be challenging in many parts of the world where human activities have disrupted wildlife communities through extinctions and introductions, every effort should be made to minimise such ecological and evolutionary loss in the World Heritage Site and Biosphere Reserve that is the Galápagos Islands.

## Supporting Information

S1 Fig Neighbour joining phylogenetic tree based on the 18S sequences. The percentage of bootstrap support (based on 1000 replicates) is indicated on each node of the tree. A bar indicates the number of substitution changes according to the Kimura 2-parameters genetic distance. Galapagos tortoise *Actractis*-like specimens sequences: 453-18-S F, 539-1-18S F, 1281-2-18S F, 1281-3-18S F, N5-1-18S F, N5-2-18S F, P-1-18S F, SR005 8-Nem18-S-F, S/N-EI.07, 117 9-Nem 18-S-F, SR001 6-Nem 18-S-F; Galapagos tortoise *Labiduris*-like specimen sequences: 453 3-Nem18-S-F, 454 1-Nem18-S-F.
(TIF)

S1 Table Number of eggs counted per gram of faeces for each sample and egg type. Trich: Trichurid; Asc: Ascarid; Oxyur: Oxyurid; Small st: Small strongyle; Large st: Large strongyle; Undet: Undetermined; BC: Breeding centre; Juv: Juvenile.
(DOCX)
