# Supplementary material for: Biogeography of Parasitic Nematode Communities in the Galápagos Giant Tortoise: Implications for Conservation Management
Source: PLoS One. 2015 Sep 2;10(9):e0135684. doi: 10.1371/journal.pone.0135684 (PMC4567182; doi:10.1371/journal.pone.0135684)
Supplement: S1 Table — Trich: Trichurid; Asc: Ascarid; Oxyur: Oxyurid; Small st: Small strongyle; Large st: Large strongyle; Undet: Undetermined; BC: Breeding centre; Juv: Juvenile. (DOCX) [file pone.0135684.s002.docx]

**S1 Table. Number of eggs counted per gram of faeces for each sample and egg type.**

| **Location** | **Sex** | **Age** | **Trich.** | **Asc.** | **Oxyur.** | **Small st.** | **Large st.** | **Undet.** |
| --- | --- | --- | --- | --- | --- | --- | --- | --- |
| Santa Cruz | Female | Adult | 0 | 0 | 0 | 0 | 920 | 0 |
| Santa Cruz |  |  | 0 | 0 | 0 | 0 | 690 | 0 |
| Santa Cruz | Female | Adult | 0 | 10 | 20 | 0 | 580 | 0 |
| Santa Cruz |  |  | 0 | 0 | 0 | 0 | 500 | 0 |
| Santa Cruz |  |  | 0 | 0 | 0 | 0 | 450 | 0 |
| Santa Cruz |  | Juvenile | 0 | 0 | 10 | 0 | 400 | 20 |
| Santa Cruz |  |  | 0 | 0 | 20 | 0 | 400 | 0 |
| Santa Cruz | Male | Adult | 0 | 0 | 60 | 0 | 340 | 10 |
| Santa Cruz | Male | Adult | 0 | 0 | 0 | 0 | 300 | 0 |
| Santa Cruz |  |  | 0 | 0 | 10 | 0 | 260 | 0 |
| Santa Cruz | Male | Adult | 0 | 0 | 20 | 0 | 250 | 0 |
| Santa Cruz |  |  | 0 | 0 | 0 | 0 | 250 | 0 |
| Santa Cruz | Male | Adult | 0 | 0 | 0 | 0 | 230 | 0 |
| Santa Cruz |  | Juvenile | 0 | 10 | 0 | 0 | 190 | 0 |
| Santa Cruz | Male | Adult | 0 | 0 | 10 | 0 | 180 | 0 |
| Santa Cruz | Female | Adult | 0 | 20 | 0 | 0 | 170 | 0 |
| Santa Cruz | Female | Adult | 0 | 0 | 0 | 0 | 160 | 0 |
| Santa Cruz |  |  | 0 | 0 | 0 | 0 | 140 | 0 |
| Santa Cruz | Female | Adult | 0 | 10 | 10 | 0 | 110 | 0 |
| Santa Cruz |  |  | 0 | 0 | 0 | 0 | 120 | 0 |
| Santa Cruz | Female | Adult | 0 | 10 | 0 | 0 | 110 | 0 |
| Santa Cruz | Male | Adult | 0 | 0 | 10 | 0 | 110 | 0 |
| Santa Cruz |  |  | 0 | 0 | 0 | 0 | 110 | 0 |
| Santa Cruz | Male | Adult | 0 | 10 | 10 | 0 | 80 | 10 |
| Santa Cruz | Female | Adult | 0 | 0 | 0 | 0 | 110 | 0 |
| Santa Cruz | Male | Adult | 0 | 0 | 0 | 0 | 100 | 0 |
| Santa Cruz | Male | Adult | 0 | 0 | 20 | 0 | 80 | 0 |
| Santa Cruz |  |  | 0 | 0 | 0 | 0 | 100 | 0 |
| Santa Cruz | Male | Adult | 0 | 0 | 0 | 0 | 90 | 0 |
| Santa Cruz | Male | Adult | 0 | 10 | 0 | 0 | 70 | 0 |
| Santa Cruz |  |  | 0 | 0 | 0 | 0 | 80 | 0 |
| Santa Cruz | Male | Adult | 0 | 10 | 0 | 0 | 70 | 0 |
| Santa Cruz |  | Juvenile | 0 | 0 | 0 | 0 | 80 | 0 |
| Santa Cruz |  |  | 0 | 0 | 0 | 0 | 70 | 0 |
| Santa Cruz |  |  | 0 | 0 | 0 | 0 | 70 | 0 |
| Santa Cruz | Female | Adult | 0 | 0 | 0 | 0 | 70 | 0 |
| Santa Cruz | Female | Adult | 0 | 0 | 0 | 0 | 70 | 0 |
| Santa Cruz |  | Juvenile | 20 | 20 | 0 | 0 | 10 | 20 |
| Santa Cruz | Female | Adult | 0 | 0 | 0 | 0 | 70 | 0 |
| Santa Cruz | Male | Adult | 0 | 10 | 0 | 0 | 60 | 0 |
| Santa Cruz | Male | Adult | 0 | 30 | 0 | 0 | 40 | 0 |
| Santa Cruz |  |  | 0 | 0 | 10 | 0 | 50 | 0 |
| Santa Cruz |  |  | 0 | 0 | 0 | 0 | 60 | 0 |
| Santa Cruz | Female | Adult | 0 | 0 | 0 | 0 | 60 | 0 |
| Santa Cruz |  |  | 0 | 0 | 0 | 0 | 60 | 0 |
| Santa Cruz |  |  | 0 | 0 | 0 | 0 | 60 | 0 |
| Santa Cruz | Male | Adult | 0 | 0 | 0 | 0 | 50 | 0 |
| Santa Cruz |  |  | 0 | 0 | 0 | 0 | 50 | 0 |
| Santa Cruz |  |  | 0 | 0 | 0 | 0 | 50 | 0 |
| Santa Cruz |  |  | 0 | 0 | 0 | 0 | 50 | 0 |
| Santa Cruz | Male | Adult | 0 | 0 | 10 | 0 | 20 | 10 |
| Santa Cruz | Female | Adult | 0 | 0 | 0 | 0 | 40 | 0 |
| Santa Cruz | Male | Adult | 0 | 0 | 0 | 0 | 40 | 0 |
| Santa Cruz | Male | Adult | 0 | 0 | 0 | 0 | 40 | 0 |
| Santa Cruz |  |  | 0 | 0 | 0 | 0 | 40 | 0 |
| Santa Cruz |  |  | 0 | 0 | 0 | 0 | 40 | 0 |
| Santa Cruz | Female | Adult | 0 | 0 | 0 | 0 | 40 | 0 |
| Santa Cruz |  | Juvenile | 0 | 0 | 0 | 0 | 40 | 0 |
| Santa Cruz | Male | Adult | 0 | 0 | 0 | 0 | 30 | 0 |
| Santa Cruz |  |  | 0 | 0 | 0 | 0 | 30 | 0 |
| Santa Cruz | Male | Adult | 0 | 0 | 0 | 0 | 30 | 0 |
| Santa Cruz |  |  | 0 | 0 | 0 | 0 | 30 | 0 |
| Santa Cruz |  |  | 0 | 0 | 0 | 0 | 30 | 0 |
| Santa Cruz | Male | Adult | 0 | 0 | 0 | 0 | 30 | 0 |
| Santa Cruz | Male | Adult | 0 | 0 | 0 | 0 | 30 | 0 |
| Santa Cruz | Male | Adult | 0 | 0 | 0 | 0 | 30 | 0 |
| Santa Cruz |  |  | 0 | 0 | 0 | 0 | 20 | 0 |
| Santa Cruz |  |  | 0 | 0 | 0 | 0 | 20 | 0 |
| Santa Cruz |  |  | 0 | 0 | 0 | 0 | 20 | 0 |
| Santa Cruz |  |  | 0 | 0 | 0 | 0 | 20 | 0 |
| Santa Cruz |  |  | 0 | 0 | 0 | 0 | 20 | 0 |
| Santa Cruz |  |  | 0 | 0 | 0 | 0 | 20 | 0 |
| Santa Cruz |  |  | 0 | 0 | 0 | 0 | 20 | 0 |
| Santa Cruz |  |  | 0 | 0 | 0 | 0 | 20 | 0 |
| Santa Cruz |  |  | 0 | 0 | 0 | 0 | 20 | 0 |
| Santa Cruz |  |  | 0 | 0 | 0 | 0 | 20 | 0 |
| Santa Cruz | Male | Adult | 0 | 0 | 0 | 0 | 20 | 0 |
| Santa Cruz | Male | Adult | 0 | 0 | 0 | 0 | 20 | 0 |
| Santa Cruz | Male | Adult | 0 | 0 | 0 | 0 | 20 | 0 |
| Santa Cruz | Male | Adult | 0 | 0 | 0 | 0 | 20 | 0 |
| Santa Cruz | Male | Adult | 0 | 0 | 0 | 0 | 20 | 0 |
| Santa Cruz | Male | Adult | 0 | 0 | 0 | 0 | 10 | 10 |
| Santa Cruz | Male | Adult | 0 | 0 | 10 | 0 | 0 | 0 |
| Santa Cruz |  |  | 0 | 0 | 0 | 0 | 10 | 0 |
| Santa Cruz |  |  | 0 | 0 | 0 | 0 | 10 | 0 |
| Santa Cruz |  |  | 0 | 0 | 0 | 0 | 10 | 0 |
| Santa Cruz |  |  | 0 | 0 | 0 | 0 | 10 | 0 |
| Santa Cruz |  |  | 0 | 0 | 0 | 0 | 10 | 0 |
| Santa Cruz |  |  | 0 | 0 | 0 | 0 | 10 | 0 |
| Santa Cruz |  |  | 0 | 0 | 0 | 0 | 10 | 0 |
| Santa Cruz |  |  | 0 | 0 | 0 | 0 | 10 | 0 |
| Santa Cruz | Male | Adult | 0 | 0 | 0 | 0 | 10 | 0 |
| Santa Cruz | Female | Adult | 0 | 0 | 0 | 0 | 10 | 0 |
| Santa Cruz | Female | Adult | 0 | 0 | 0 | 0 | 10 | 0 |
| Santa Cruz |  |  | 0 | 0 | 0 | 0 | 10 | 0 |
| Santa Cruz |  |  | 0 | 0 | 0 | 0 | 10 | 0 |
| Santa Cruz |  |  | 0 | 0 | 0 | 0 | 10 | 0 |
| Santa Cruz | Male | Adult | 0 | 0 | 0 | 0 | 10 | 0 |
| Santa Cruz |  |  | 0 | 0 | 0 | 0 | 10 | 0 |
| Santa Cruz | Male | Adult | 0 | 0 | 0 | 0 | 10 | 0 |
| Santa Cruz |  |  | 0 | 0 | 0 | 0 | 0 | 10 |
| Santa Cruz |  |  | 0 | 0 | 0 | 0 | 10 | 0 |
| Santa Cruz | Male | Adult | 0 | 0 | 0 | 0 | 10 | 0 |
| Santa Cruz | Male | Adult | 0 | 0 | 0 | 0 | 0 | 0 |
| Santa Cruz | Female | Adult | 0 | 0 | 0 | 0 | 0 | 0 |
| Santa Cruz |  |  | 0 | 0 | 0 | 0 | 0 | 0 |
| Santa Cruz |  |  | 0 | 0 | 0 | 0 | 0 | 0 |
| Santa Cruz |  |  | 0 | 0 | 0 | 0 | 0 | 0 |
| Santa Cruz |  |  | 0 | 0 | 0 | 0 | 0 | 0 |
| Santa Cruz |  |  | 0 | 0 | 0 | 0 | 0 | 0 |
| Santa Cruz |  |  | 0 | 0 | 0 | 0 | 0 | 0 |
| Santa Cruz |  |  | 0 | 0 | 0 | 0 | 0 | 0 |
| Santa Cruz |  |  | 0 | 0 | 0 | 0 | 0 | 0 |
| Santa Cruz |  |  | 0 | 0 | 0 | 0 | 0 | 0 |
| Santa Cruz |  |  | 0 | 0 | 0 | 0 | 0 | 0 |
| Santa Cruz |  |  | 0 | 0 | 0 | 0 | 0 | 0 |
| Santa Cruz | Male | Adult | 0 | 0 | 0 | 0 | 0 | 0 |
| Santa Cruz | Male | Adult | 0 | 0 | 0 | 0 | 0 | 0 |
| Santa Cruz |  |  | 0 | 0 | 0 | 0 | 0 | 0 |
| Santa Cruz |  |  | 0 | 0 | 0 | 0 | 0 | 0 |
| Santa Cruz |  |  | 0 | 0 | 0 | 0 | 0 | 0 |
| Santa Cruz |  |  | 0 | 0 | 0 | 0 | 0 | 0 |
| Santa Cruz |  |  | 0 | 0 | 0 | 0 | 0 | 0 |
| Santa Cruz | Male | Adult | 0 | 0 | 0 | 0 | 0 | 0 |
| Santa Cruz |  |  | 0 | 0 | 0 | 0 | 0 | 0 |
| Santa Cruz | Male | Adult | 0 | 0 | 0 | 0 | 0 | 0 |
| Pinzón | Female | Adult | 0 | 0 | 0 | 0 | 0 | 0 |
| Pinzón | Female | Adult | 0 | 40 | 0 | 0 | 30 | 0 |
| Pinzón |  | Juvenile | 0 | 0 | 0 | 0 | 30 | 0 |
| Pinzón | Female | Adult | 0 | 0 | 0 | 0 | 0 | 0 |
| Pinzón | Male | Adult | 0 | 0 | 0 | 0 | 40 | 0 |
| Pinzón | Female | Adult | 0 | 0 | 0 | 0 | 80 | 0 |
| Pinzón | Female | Adult | 0 | 0 | 0 | 0 | 160 | 0 |
| Pinzón | Female | Adult | 0 | 0 | 0 | 0 | 40 | 0 |
| Pinzón |  | Juvenile | 0 | 0 | 0 | 0 | 10 | 0 |
| Pinzón | Male | Adult | 0 | 10 | 0 | 0 | 40 | 0 |
| Pinzón | Female | Adult | 0 | 40 | 0 | 0 | 140 | 0 |
| Pinzón | Female | Adult | 0 | 10 | 0 | 0 | 70 | 0 |
| Pinzón | Female | Adult | 0 | 0 | 0 | 0 | 20 | 0 |
| Pinzón | Female | Adult | 0 | 10 | 0 | 0 | 170 | 0 |
| Pinzón | Female | Adult | 0 | 0 | 0 | 0 | 40 | 0 |
| Pinzón | Male | Adult | 0 | 0 | 0 | 0 | 310 | 0 |
| Pinzón |  | Juvenile | 0 | 0 | 0 | 0 | 0 | 0 |
| Pinzón | Female | Adult | 0 | 0 | 0 | 0 | 80 | 0 |
| Pinzón | Male | Adult | 0 | 10 | 0 | 0 | 40 | 0 |
| Pinzón | Male | Adult | 0 | 0 | 0 | 0 | 60 | 0 |
| Pinzón | Female | Adult | 0 | 10 | 0 | 0 | 140 | 0 |
| Pinzón | Female | Adult | 0 | 30 | 0 | 0 | 40 | 0 |
| Pinzón | Female | Adult | 0 | 10 | 0 | 0 | 10 | 0 |
| Pinzón | Male | Adult | 0 | 0 | 0 | 0 | 20 | 0 |
| Pinzón | Female | Adult | 0 | 0 | 0 | 0 | 200 | 0 |
| Pinzón | Male | Adult | 0 | 0 | 0 | 0 | 10 | 0 |
| Pinzón |  | Juvenile | 0 | 0 | 0 | 0 | 90 | 0 |
| Pinzón | Female | Adult | 0 | 10 | 0 | 0 | 100 | 0 |
| Pinzón | Male | Adult | 0 | 30 | 0 | 0 | 60 | 0 |
| Pinzón | Female | Adult | 0 | 0 | 0 | 0 | 160 | 0 |
| Pinzón | Female | Adult | 0 | 0 | 0 | 0 | 0 | 0 |
| Pinzón | Male | Adult | 0 | 0 | 0 | 0 | 310 | 0 |
| Pinzón | Male | Adult | 0 | 0 | 0 | 0 | 20 | 0 |
| Pinzón | Female | Adult | 0 | 0 | 0 | 0 | 90 | 0 |
| San Pedro |  | Juvenile | 0 | 10 | 0 | 90 | 0 | 0 |
| San Pedro |  |  | 0 | 0 | 0 | 110 | 0 | 0 |
| San Pedro | Male | Adult | 0 | 0 | 30 | 130 | 0 | 0 |
| San Pedro | Male | Adult | 0 | 0 | 0 | 20 | 0 | 0 |
| San Pedro | Male | Adult | 0 | 0 | 0 | 30 | 10 | 0 |
| San Pedro |  | Juvenile | 0 | 0 | 0 | 20 | 0 | 0 |
| San Pedro | Male | Adult | 0 | 0 | 0 | 80 | 0 | 0 |
| San Pedro |  |  | 0 | 0 | 0 | 70 | 10 | 0 |
| San Pedro |  | Juvenile | 0 | 0 | 0 | 60 | 0 | 0 |
| San Pedro |  |  | 0 | 0 | 0 | 10 | 0 | 0 |
| San Pedro | Male | Adult | 0 | 0 | 0 | 170 | 0 | 0 |
| San Pedro |  | Juvenile | 10 | 10 | 0 | 10 | 0 | 0 |
| San Pedro |  | Juvenile | 0 | 0 | 0 | 150 | 0 | 0 |
| San Pedro |  | Juvenile | 0 | 0 | 0 | 0 | 0 | 0 |
| San Pedro |  | Juvenile | 0 | 0 | 0 | 0 | 0 | 0 |
| San Pedro | Male | Adult | 0 | 0 | 0 | 40 | 0 | 0 |
| San Pedro |  | Juvenile | 0 | 0 | 0 | 130 | 0 | 0 |
| San Pedro | Male | Adult | 0 | 0 | 0 | 20 | 0 | 0 |
| San Pedro |  | Juvenile | 0 | 0 | 0 | 0 | 0 | 0 |
| San Pedro |  | Juvenile | 0 | 0 | 0 | 30 | 0 | 0 |
| San Pedro |  | Juvenile | 0 | 0 | 0 | 0 | 0 | 0 |
| San Pedro | Male | Adult | 0 | 0 | 0 | 20 | 0 | 0 |
| Roca Union | Female | Adult | 0 | 0 | 0 | 180 | 80 | 10 |
| Roca Union | Female | Adult | 0 | 0 | 0 | 50 | 10 | 0 |
| Roca Union | Female | Adult | 0 | 0 | 0 | 0 | 0 | 0 |
| Roca Union | Female | Adult | 0 | 10 | 0 | 100 | 0 | 20 |
| Roca Union |  | Juvenile | 0 | 0 | 0 | 0 | 0 | 0 |
| Roca Union | Female | Adult | 30 | 0 | 0 | 20 | 0 | 0 |
| Roca Union | Male | Adult | 0 | 0 | 0 | 450 | 0 | 0 |
| Roca Union | Female | Adult | 0 | 0 | 0 | 10 | 0 | 0 |
| Roca Union |  | Juvenile | 0 | 0 | 0 | 30 | 20 | 0 |
| Roca Union | Female | Adult | 0 | 0 | 0 | 10 | 0 | 0 |
| Roca Union | Female | Adult | 0 | 0 | 0 | 600 | 0 | 0 |
| Roca Union | Female | Adult | 0 | 10 | 0 | 400 | 0 | 0 |
| Roca Union |  | Juvenile | 20 | 30 | 0 | 740 | 0 | 0 |
| Roca Union |  | Juvenile | 30 | 0 | 0 | 0 | 0 | 0 |
| Roca Union |  |  | 0 | 0 | 0 | 80 | 0 | 0 |
| Roca Union | Female | Adult | 0 | 0 | 0 | 410 | 0 | 0 |
| Roca Union | Female | Adult | 0 | 0 | 20 | 90 | 0 | 0 |
| Roca Union | Male | Adult | 0 | 0 | 0 | 100 | 0 | 0 |
| Roca Union |  | Juvenile | 10 | 0 | 0 | 1140 | 0 | 0 |
| Roca Union |  | Juvenile | 0 | 0 | 0 | 4150 | 0 | 0 |
| Roca Union |  | Juvenile | 0 | 0 | 0 | 250 | 0 | 0 |
| Roca Union |  | Juvenile | 0 | 0 | 20 | 1000 | 0 | 0 |
| Roca Union |  | Juvenile | 20 | 0 | 0 | 20 | 0 | 0 |
| Roca Union |  | Juvenile | 0 | 0 | 0 | 150 | 0 | 0 |
| Roca Union |  |  | 0 | 0 | 0 | 110 | 0 | 0 |
| Santa Cruz BC | Female | Adult | 0 | 0 | 0 | 0 | 0 | 0 |
| Santa Cruz BC | Female | Adult | 0 | 0 | 0 | 0 | 50 | 0 |
| Santa Cruz BC | Male | Adult | 0 | 0 | 0 | 0 | 40 | 0 |
| Santa Cruz BC | Female | Adult | 0 | 0 | 0 | 0 | 40 | 0 |
| Santa Cruz BC | Female | Adult | 0 | 0 | 0 | 0 | 40 | 0 |
| Santa Cruz BC | Female | Adult | 0 | 0 | 0 | 0 | 0 | 0 |
| Santa Cruz BC | Female | Adult | 0 | 0 | 0 | 0 | 10 | 0 |
| Santa Cruz BC | Female | Adult | 0 | 0 | 0 | 0 | 20 | 0 |
| Santa Cruz BC | Female | Adult | 0 | 0 | 0 | 0 | 30 | 0 |
| Santa Cruz BC | Female | Adult | 0 | 0 | 0 | 0 | 40 | 0 |
| Santa Cruz BC | Female | Adult | 0 | 0 | 0 | 0 | 0 | 0 |
| Santa Cruz BC | Female | Adult | 0 | 0 | 0 | 0 | 30 | 0 |
| Santa Cruz BC | Male | Adult | 0 | 0 | 30 | 0 | 190 | 0 |
| Santa Cruz BC | Male | Adult | 0 | 0 | 0 | 0 | 0 | 0 |
| Santa Cruz BC | Male | Adult | 0 | 0 | 0 | 0 | 20 | 0 |
| Santa Cruz BC | Male | Adult | 0 | 0 | 0 | 0 | 0 | 0 |
| Santa Cruz BC | Male | Adult | 0 | 0 | 0 | 0 | 20 | 0 |
| Santa Cruz BC | Male | Adult | 0 | 0 | 0 | 0 | 160 | 0 |
| Santa Cruz BC | Male | Adult | 0 | 0 | 0 | 0 | 20 | 0 |
| Santa Cruz BC | Male | Adult | 0 | 0 | 0 | 0 | 90 | 0 |
| Santa Cruz BC | Male | Adult | 0 | 0 | 0 | 0 | 60 | 0 |
| Santa Cruz BC | Male | Adult | 0 | 0 | 0 | 0 | 20 | 0 |
| Santa Cruz BC | Male | Adult | 0 | 0 | 0 | 0 | 0 | 0 |
| Santa Cruz BC | Female | Adult | 0 | 10 | 0 | 0 | 50 | 0 |
| Santa Cruz BC | Male | Adult | 0 | 10 | 0 | 0 | 20 | 0 |
| Santa Cruz BC | Male | Adult | 0 | 0 | 0 | 0 | 0 | 0 |
| Santa Cruz BC | Female | Adult | 0 | 0 | 0 | 0 | 10 | 0 |
| Santa Cruz BC | Female | Adult | 0 | 10 | 0 | 0 | 150 | 0 |
| Santa Cruz BC | Female | Adult | 0 | 10 | 0 | 0 | 110 | 0 |
| Santa Cruz BC | Female | Adult | 0 | 0 | 0 | 0 | 0 | 0 |
| Santa Cruz BC | Female | Adult | 0 | 0 | 0 | 0 | 0 | 0 |
| Santa Cruz BC | Female | Adult | 0 | 0 | 0 | 0 | 0 | 0 |
| Santa Cruz BC | Male | Adult | 0 | 0 | 0 | 0 | 0 | 0 |
| Santa Cruz BC | Female | Adult | 0 | 0 | 0 | 0 | 0 | 0 |
| Santa Cruz BC | Female | Adult | 0 | 0 | 0 | 0 | 0 | 0 |
| Santa Cruz BC | Male | Adult | 0 | 0 | 0 | 0 | 0 | 0 |
| Santa Cruz BC | Female | Adult | 0 | 0 | 0 | 0 | 0 | 0 |
| Santa Cruz BC | Female | Adult | 0 | 0 | 0 | 0 | 0 | 0 |
| Santa Cruz BC | Female | Adult | 0 | 0 | 0 | 0 | 40 | 0 |
| Santa Cruz BC | Male | Adult | 0 | 0 | 0 | 0 | 20 | 0 |
| Santa Cruz BC | Male | Adult | 0 | 0 | 0 | 0 | 40 | 0 |
| Santa Cruz BC | Male | Adult | 0 | 0 | 0 | 0 | 50 | 0 |
| Santa Cruz BC | Male | Adult | 0 | 0 | 0 | 0 | 0 | 0 |
| Santa Cruz BC | Male | Adult | 0 | 0 | 0 | 0 | 0 | 0 |
| Santa Cruz BC | Male | Adult | 0 | 0 | 0 | 0 | 30 | 0 |
| Santa Cruz BC | Male | Adult | 0 | 0 | 0 | 0 | 0 | 0 |
| Santa Cruz BC | Male | Adult | 0 | 0 | 0 | 0 | 0 | 0 |
| Santa Cruz BC | Male | Adult | 0 | 0 | 0 | 0 | 0 | 0 |
| Santa Cruz BC | Male | Adult | 0 | 0 | 0 | 0 | 0 | 0 |
| Santa Cruz BC | Male | Adult | 0 | 0 | 0 | 0 | 0 | 0 |
| Santa Cruz BC | Male | Adult | 0 | 0 | 0 | 0 | 60 | 0 |
| Santa Cruz BC | Male | Adult | 0 | 0 | 0 | 0 | 10 | 0 |
| Santa Cruz BC | Male | Adult | 0 | 0 | 0 | 0 | 100 | 0 |
| Santa Cruz BC | Male | Adult | 0 | 0 | 0 | 0 | 0 | 0 |
| Santa Cruz BC | Female | Adult | 0 | 0 | 0 | 0 | 30 | 0 |
| Santa Cruz BC | Female | Adult | 0 | 0 | 0 | 0 | 10 | 0 |
| Santa Cruz BC | Female | Adult | 0 | 0 | 0 | 0 | 140 | 0 |
| Santa Cruz BC | Female | Adult | 0 | 0 | 0 | 0 | 30 | 0 |
| Santa Cruz BC | Male | Adult | 0 | 0 | 0 | 0 | 0 | 0 |
| Santa Cruz BC | Male | Adult | 0 | 0 | 0 | 0 | 10 | 0 |
| Santa Cruz BC | Male | Adult | 0 | 0 | 0 | 0 | 0 | 0 |
| Santa Cruz BC | Male | Adult | 0 | 0 | 0 | 0 | 0 | 0 |
| Santa Cruz BC | Female | Adult | 0 | 0 | 0 | 0 | 160 | 0 |
| Santa Cruz BC | Female | Adult | 0 | 0 | 0 | 0 | 150 | 0 |
| Santa Cruz BC | Female | Adult | 0 | 0 | 0 | 0 | 90 | 0 |
| Santa Cruz BC Juv |  | Juvenile | 0 | 0 | 0 | 0 | 80 | 0 |
| Santa Cruz BC Juv |  | Juvenile | 0 | 0 | 0 | 0 | 0 | 0 |
| Santa Cruz BC Juv |  | Juvenile | 0 | 0 | 0 | 0 | 30 | 0 |
| Santa Cruz BC Juv |  | Juvenile | 0 | 0 | 0 | 0 | 0 | 0 |
| Santa Cruz BC Juv |  | Juvenile | 0 | 0 | 0 | 0 | 10 | 0 |
| Santa Cruz BC Juv |  | Juvenile | 0 | 0 | 0 | 0 | 0 | 0 |
| Santa Cruz BC Juv |  | Juvenile | 0 | 0 | 0 | 0 | 0 | 0 |
| Santa Cruz BC Juv |  | Juvenile | 0 | 0 | 0 | 0 | 0 | 0 |
| Santa Cruz BC Juv |  | Juvenile | 0 | 0 | 0 | 0 | 10 | 0 |
| Santa Cruz BC Juv |  | Juvenile | 0 | 0 | 0 | 0 | 0 | 0 |
| Santa Cruz BC Juv |  | Juvenile | 0 | 0 | 0 | 0 | 0 | 0 |
| Santa Cruz BC Juv |  | Juvenile | 0 | 0 | 0 | 0 | 0 | 0 |
| Santa Cruz BC Juv |  | Juvenile | 0 | 0 | 0 | 0 | 0 | 0 |
| Santa Cruz BC Juv |  | Juvenile | 0 | 0 | 0 | 0 | 0 | 0 |
| Santa Cruz BC Juv |  | Juvenile | 0 | 0 | 0 | 0 | 0 | 0 |
| Santa Cruz BC Juv |  | Juvenile | 0 | 0 | 0 | 0 | 0 | 0 |
| Santa Cruz BC Juv |  | Juvenile | 0 | 0 | 0 | 0 | 0 | 0 |
| Santa Cruz BC Juv |  | Juvenile | 0 | 0 | 0 | 0 | 0 | 0 |
| Santa Cruz BC Juv |  | Juvenile | 0 | 0 | 0 | 0 | 0 | 0 |
| Santa Cruz BC Juv |  | Juvenile | 0 | 0 | 0 | 0 | 0 | 0 |
| Santa Cruz BC Juv |  | Juvenile | 0 | 0 | 0 | 0 | 0 | 0 |
| Santa Cruz BC Juv |  | Juvenile | 0 | 0 | 0 | 0 | 10 | 0 |
| Santa Cruz BC Juv |  | Juvenile | 0 | 0 | 0 | 0 | 10 | 0 |
| Santa Cruz BC Juv |  | Juvenile | 0 | 0 | 0 | 0 | 0 | 0 |
| Santa Cruz BC Juv |  | Juvenile | 0 | 0 | 0 | 0 | 0 | 0 |
| Santa Cruz BC Juv |  | Juvenile | 0 | 0 | 0 | 0 | 0 | 0 |
| Santa Cruz BC Juv |  | Juvenile | 0 | 0 | 0 | 0 | 0 | 0 |
| Santa Cruz BC Juv |  | Juvenile | 0 | 0 | 0 | 0 | 0 | 0 |
| Santa Cruz BC Juv |  | Juvenile | 0 | 0 | 0 | 0 | 10 | 0 |
| Santa Cruz BC Juv |  | Juvenile | 0 | 0 | 0 | 0 | 0 | 0 |
| Santa Cruz BC Juv |  | Juvenile | 0 | 0 | 0 | 0 | 0 | 0 |
| Santa Cruz BC Juv |  | Juvenile | 0 | 0 | 0 | 0 | 10 | 0 |
| Isabela BC |  | Adult | 0 | 0 | 0 | 0 | 0 | 0 |
| Isabela BC |  | Adult | 0 | 0 | 0 | 0 | 0 | 0 |
| Isabela BC |  | Adult | 0 | 0 | 0 | 0 | 0 | 0 |
| Isabela BC |  | Adult | 0 | 0 | 0 | 0 | 0 | 0 |
| Isabela BC |  | Adult | 0 | 0 | 0 | 0 | 0 | 0 |
| Isabela BC |  | Adult | 0 | 0 | 0 | 0 | 0 | 0 |
| Isabela BC |  | Adult | 0 | 0 | 0 | 0 | 0 | 0 |
| Isabela BC |  | Adult | 0 | 0 | 0 | 0 | 0 | 0 |
| Isabela BC |  | Adult | 0 | 0 | 0 | 0 | 0 | 0 |
| Isabela BC |  | Adult | 0 | 0 | 0 | 0 | 0 | 0 |
| Isabela BC |  | Adult | 0 | 0 | 0 | 0 | 0 | 0 |
| Isabela BC |  | Adult | 0 | 0 | 0 | 0 | 0 | 0 |
| Isabela BC |  | Adult | 0 | 0 | 0 | 0 | 0 | 0 |
| Isabela BC |  | Adult | 0 | 0 | 0 | 0 | 0 | 0 |
| Isabela BC |  | Adult | 0 | 0 | 0 | 0 | 0 | 0 |
| Isabela BC |  | Adult | 0 | 0 | 0 | 0 | 0 | 0 |
| Isabela BC |  | Adult | 0 | 0 | 0 | 0 | 0 | 0 |
| Isabela BC |  | Adult | 0 | 0 | 0 | 0 | 0 | 0 |
| Isabela BC |  | Adult | 0 | 0 | 0 | 0 | 0 | 0 |
| Isabela BC |  | Adult | 0 | 10 | 0 | 230 | 200 | 0 |
| Isabela BC |  | Adult | 0 | 0 | 0 | 40 | 10 | 0 |
| Isabela BC Juv |  | Juvenile | 0 | 0 | 0 | 0 | 0 | 0 |
| Isabela BC Juv |  | Juvenile | 0 | 0 | 0 | 0 | 0 | 0 |
| Isabela BC Juv |  | Juvenile | 0 | 0 | 0 | 0 | 0 | 0 |
| Isabela BC Juv |  | Juvenile | 0 | 0 | 0 | 0 | 0 | 0 |
| Isabela BC Juv |  | Juvenile | 0 | 0 | 0 | 0 | 0 | 0 |
| Isabela BC Juv |  | Juvenile | 0 | 0 | 0 | 0 | 0 | 0 |
| Isabela BC Juv |  | Juvenile | 0 | 0 | 0 | 0 | 0 | 0 |
| Isabela BC Juv |  | Juvenile | 0 | 0 | 0 | 0 | 0 | 0 |
| Isabela BC Juv |  | Juvenile | 0 | 0 | 0 | 0 | 20 | 0 |
| Isabela BC Juv |  | Juvenile | 0 | 0 | 0 | 0 | 0 | 0 |
| San Cristobal BC |  | Adult | 0 | 0 | 0 | 0 | 0 | 0 |
| San Cristobal BC |  | Adult | 0 | 0 | 0 | 0 | 0 | 0 |
| San Cristobal BC |  | Adult | 0 | 0 | 0 | 0 | 0 | 0 |
| San Cristobal BC |  | Adult | 0 | 0 | 0 | 0 | 0 | 0 |
| San Cristobal BC |  | Adult | 0 | 0 | 0 | 0 | 0 | 0 |
| San Cristobal BC |  | Adult | 0 | 0 | 0 | 0 | 0 | 0 |
| San Cristobal BC |  | Adult | 0 | 0 | 0 | 0 | 0 | 0 |
| San Cristobal BC |  | Adult | 0 | 0 | 0 | 0 | 0 | 0 |
| San Cristobal BC |  | Adult | 0 | 0 | 0 | 0 | 0 | 0 |
| San Cristobal BC |  | Adult | 0 | 0 | 0 | 0 | 0 | 0 |
| San Cristobal BC |  | Adult | 0 | 0 | 0 | 0 | 0 | 0 |
| San Cristobal BC |  | Adult | 0 | 0 | 0 | 0 | 0 | 0 |
| San Cristobal BC |  | Adult | 0 | 0 | 0 | 0 | 0 | 0 |
| San Cristobal BC |  | Adult | 0 | 0 | 0 | 0 | 0 | 0 |
| San Cristobal BC |  | Adult | 0 | 0 | 0 | 0 | 0 | 0 |
| San Cristobal BC |  | Adult | 0 | 0 | 0 | 0 | 0 | 0 |
| San Cristobal BC |  | Adult | 0 | 0 | 0 | 0 | 0 | 0 |
| San Cristobal BC |  | Adult | 0 | 0 | 0 | 0 | 0 | 0 |
| San Cristobal BC |  | Adult | 0 | 0 | 0 | 0 | 0 | 0 |
| San Cristobal BC |  | Adult | 0 | 0 | 0 | 0 | 0 | 0 |
| San Cristobal BC |  | Adult | 0 | 0 | 0 | 0 | 0 | 0 |
| San Cristobal BC |  | Adult | 0 | 0 | 0 | 0 | 0 | 0 |
| San Cristobal BC |  | Adult | 0 | 0 | 0 | 0 | 0 | 0 |
| San Cristobal BC |  | Adult | 0 | 0 | 0 | 0 | 0 | 0 |
| San Cristobal BC |  | Adult | 0 | 0 | 0 | 0 | 0 | 0 |
| San Cristobal BC |  | Adult | 0 | 0 | 0 | 0 | 0 | 0 |
| San Cristobal BC |  | Adult | 0 | 0 | 0 | 0 | 0 | 0 |

Trich: Trichurid; Asc: Ascarid; Oxyur: Oxyurid; Small st: Small strongyle; Large st: Large strongyle; Undet: Undetermined; BC: Breeding centre; Juv: Juvenile
